# Antecedents of the Attitudes Toward Singlehood Among Young Adults in Malaysia, Japan, and India

**DOI:** 10.3389/fpsyg.2021.756090

**Published:** 2021-11-19

**Authors:** Chee-Seng Tan, Siew-May Cheng, Tomokazu Nakayama, Sanju George

**Affiliations:** ^1^Department of Psychology and Counselling, Faculty of Arts and Social Science, Universiti Tunku Abdul Rahman (UTAR), Kampar, Malaysia; ^2^Department of Languages and Linguistics, Faculty of Arts and Social Science, Universiti Tunku Abdul Rahman (UTAR), Kampar, Malaysia; ^3^Center for Language Education and International Programs, Jissen Women’s University, Tokyo, Japan; ^4^Lisie Hospital, Ernakulam, India

**Keywords:** Asia, attitudes, autonomy, romantic relationship, singlehood, voluntary singlehood, young adults

## Abstract

With both theories and empirical studies supporting the benefits of having a romantic relationship, there remains an increasing tendency of staying single being documented globally. It is thus important to understand the antecedent factors of such voluntary single movement. Guided by the Investment Model of Commitment (IMC) process, the roles of subjective socioeconomic status (SSES), relational mobility, and desirability of control in attitudes toward singlehood were investigated. A total of 1,108 undergraduate students from Malaysia (*n*=444), Japan (*n*=316), and India (*n*=348) answered an online survey consisting of the Attitudes toward Singlehood Scale, MacArthur Scale of SSES, Relational Mobility Scale, Desirability of Control Scale, Mini-Social Phobia Inventory, and Single Item Narcissism Scale. Hierarchical multiple regression analysis showed a persistent positive relationship between desirability of control, but not socioeconomic status and relational mobility, with attitudes toward singlehood, even after statistically excluding the effects of social anxiety and narcissism. A similar pattern was also observed among those who were currently single. Moreover, an interaction effect of socioeconomic status and relational mobility was found in further exploratory analysis. The results highlight that retaining the autonomy and flexibility of managing one’s own life and financial concern are the key reasons young adults prefer staying single to engaging in a romantic relationship. Implications and recommendations for future research are also presented in this study.

## Introduction

As far as relationships are concerned, committed individuals that include married couples (after statistically controlling for pre-marital life satisfaction levels) are more satisfied with their lives following better well-being compared to their single counterparts (McCabe et al., 1996; Hope et al., 1999; Hudson et al., 2020). For instance, social maturity, better adjustment, and less self-centeredness are observed in previously or currently dating undergraduates than those who were currently single and always single (DePaulo and Morris, 2006). In terms of fear of being single, people found its positive relationship with loneliness (Spielmann et al., 2013) and negative relationship with (emotional and psychological) well-being and life satisfaction (Adamczyk, 2017).

To some others, getting married or being involved in romantic relationships may not necessarily promote well-being in individuals. DePaulo (2015) and DePaulo and Morris (2005, 2006) proclaimed the lack of scholarly support to the popular belief whereby getting married would guarantee assured health or lasting happiness in people. For instance, when examining the currently-married and unmarried individuals’ health conditions, the ones that appear less healthy than the currently-married are divorcees, widows, and widowers except the singles (DePaulo, 2013). Just as never-married adults equipped with higher autonomy or self-sufficiency would experience lower negative affect (Bookwala and Fekete, 2009), both top-scoring never-married and married adults in individual mastery (i.e., control of personal lives) would display a similar level of negative affects. In other words, the findings suggest that the level of individual mastery, but not marriage, is the key to affective well-being among single and married adults. In fact, single people and married people are enjoying the same levels of health and happiness (DePaulo and Morris, 2006; DePaulo, 2013).

Ironically, despite the many advantages of singlehood, the number of single young people is growing exponentially compared to that of coupled individuals (United States Census Bureau, 2017; Wu, 2017; Rich, 2019). For instance, there is a stark increase in unmarried people from 28% in 1970 to over 44% in 2012 as well as a rapid decrease in married people from about 70% in 1970 to only 49% in 2011 in the United States alone (DePaulo, 2014). According to Apostolou (2019), the common high population of singles in the Western countries has raised the issue of people staying single by choice or due to difficulties in courtship. For the latter, singles are being involuntarily single (Apostolou et al., 2019a) because of their poor mating performances (Apostolou et al., 2019b). Meanwhile, other circumstance could be that young undergraduates emphasizing more personal than social lives (Takada, 1992), for instance, are found to be staying single longer than the others (Chandler et al., 2004). In an Asian country like Malaysia, the recent number of marriages had decreased by 1.2% from 206,352 in 2018 to 203,821 in 2019 (Department of Statistics, 2020). Almost two million Malaysian women above 30years old in Selangor and Johor are unmarried (Lee, 2020).

## Attitudes Toward Singlehood

As reviewed in the literature, couples may not have enjoyed the perks of singlehood (e.g., Lehmann et al., 2015) namely autonomy, temporal control, enhanced sociability, job advancement, and non-compliance to other’s requests (Whillans, 2014). Singles during the modern industrialization era are free to alternate their social lives and ways of living (Galcanova and Vackova, 2016) besides enjoying singlehood unlike others seeking partnership (Frazier et al., 1996). Besides, singlehood is also deemed as a personal choice for privacy and alone time from bitter relationships or simply one’s preferred job, spiritual, or religious belief (Band-Winterstein and Manchik-Rimon, 2014; Timonen and Doyle, 2014).

Elsewhere, while studying social status brought by rising global singlehood, the concept of “Sampo Generation” is seen mushrooming in Korea whereby its neologism in South Korea refers to singles that decline courtship, marriage, and childbearing. With financial stability, health, and work surpassing the singles’ needs for traditional family making (Jang, 2021), a similar Korean solo-living lifestyle is also observed in other East Asian countries like Japan and China (Muradyan and Yashkina, 2020).

Recently in China, the term “Tangpingism” (躺平主义) or “Lying Flatism” went viral following a forum post on simple and economical working life of only several months in a year. Seemingly making meagre earnings in luxurious and elite cities, individuals advocating tangping, or “lying flat” lifestyle just could not endorse marriage, children, property purchase, long working hours, or simply a job. Despite Chinese officials’ loosening family-size limit rule to allow up to three children for all couples, such attitude and behaviors have just gone from bad to worse when people everywhere are only thinking about how best to lie down instead of reproducing (Kuo, 2021).

On the other hand, in Japan, a new phenomenon “herbivorization” develops among unmarried young adults, especially men, who are impassionate in finding romantic or sexual partners of the opposite sex. There is a steady increase from 27.4 to 40.7% among females and from 40.3 to 50.8% among male 18–39-year-old singles between 1992 and 2015 (Ghaznavi et al., 2020). Other than being unemployed as well as having lower income and educational qualifications, these notorious “herbivores” are expected to eventually contribute to Japan’s worsening birth rates from 127 million in 2015 to less than 90 million by 2065.

## Investment Model of Commitment Process

According to the Investment Model of Commitment (IMC) process (Rusbult et al., 1998), individuals will commit to a relationship based on three factors namely satisfaction level (SL), quality of alternatives (QoA), and investment size (IS). In other words, individuals are more intent to persist in a relationship under three conditions: when more positive than negative affects (i.e., satisfaction) exist in a relationship; there is no alternative or less desirable alternative than that in the current relationship (i.e., poor alternative); and the cost of ending the relationship such as losing resources is high (i.e., high investment). A meta-analysis on 50,427 participants from 202 independent samples shows that the satisfaction level has the strongest relationship with commitment followed by investments and QoA (Tran et al., 2019). Notably, the three predictors together explained 54% of the variance of commitment across studies on interpersonal and noninterpersonal relationships (e.g., sport and environment). The model has also been extended to online dating domain. Sharabi and Timmermans (2021) administered the revised Investment Model Scale (Rusbult et al., 1998) to 205 individuals aged 18–49 in the United States for them to self-report the alternatives, investments, satisfaction, and commitment to potential romantic mates met not physically but virtually. Analysis found that QoA had a negative relationship, while both satisfaction and investments had a positive relationship with online dating commitment. The cumulative evidence supports that IMC is a useful and promising framework to explain the motivation to commit in relationships.

## The Present Study

The IMC process has been widely used in interpersonal relationship studies to understand commitment in relationships. This study extended the IMC process to understand factors that play a role in singlehood. Grounded on the IMC process, the desirability of control, relational mobility, and subjective socioeconomic status (SSES) that act as the proxy of SL, QoA, and IS, respectively, are hypothesized to influence the attitudes toward singlehood among people.

The desirability of control refers to the “individual difference in the amount of control one wishes to have over their life outcomes” (Thomas et al., 2011, p. 173). During an informal interview conducted by the first author, undergraduates expressed their concern that they will have less control of time when they are in a relationship than in singlehood. Establishing a relationship does not bring more but less positive affects (i.e., low satisfaction) to them. In other words, desirability of control can be an indicator of satisfaction level. People who wish to have a better control of their life will be more satisfied when they are single than in a romantic relationship. It is thus hypothesized that individuals scoring higher in desirability of control are likely to be single.

Besides, relational mobility refers to casual partner seeking in specific social or societal circumstances (Yuki et al., 2007). As technological advancement increases relational mobility, individuals having more chances in meeting more people would have higher chances in seeking ideal partners, thus establishing better relationships (i.e., the QoA is higher). Put differently, high relational mobility will increase the chances for having better alternatives to a relationship and hence, individuals prefer staying single for meeting the best partner later. Thus, it is assumed that relationship mobility will have a positive relationship with attitudes toward singlehood.

On the other hand, considering that most of the undergraduate students do not have active income, this study focuses on individuals’ perception of their SSES (Heydari et al., 2013) as a proxy of financial concern. It is only reasonable to assume a negative relationship between SSES and attitudes toward singlehood since individuals with low SSES will be concerned about the increased financial burden when being in a relationship. SSES taps on the individuals’ subjective evaluation of the (financial) resources they have. While people with higher SSES have more resources (and more capability) to invest into a relationship, individuals with lower SSES tend to perceive singlehood as a better option to save their limited resources. Earlier, Petrowski et al. (2015) analyzed the data obtained from 1,676 individuals in Germany and found 71% of the singles earning less than € 2,000 monthly income compared to only 20% of partnered individuals, and 29% singles actually earning € 2,000 a month, or more compared to 80% of partnered individuals. They also discovered that individuals earning monthly income below € 2,000 were 13-fold more likely to be single than the others. This implies that the lower income people tend to be the singles who do not involve in maintaining relationships demanding high investment.

Likewise, since Apostolou et al. (2020) and Apostolou (2021) found being too picky or choosy and feeling anxious to interact with opposite-sex individuals a reason behind people staying single, it is therefore assumed that narcissism and social anxiety play a role in attitudes toward singlehood, respectively. We first measured narcissism and social anxiety then statistically excluded their effect to have a clearer picture of the relationship of SSES, relational mobility, and desirability of control with attitudes toward singlehood. The results are expected to yield more underlying reasons for young adults’ attitudes toward staying single. Moreover, this cross-cultural research done across three Asian countries could overcome the scarcity of global comparative studies on the growing trend of singlehood studies (Muradyan and Yashkina, 2020) whenever cultural variations exist (Apostolou et al., 2021). Evidently, the IMC model of Rusbult et al. (2011) has been used in studies involving non-Western samples, for instance the Chinese population in the study of Lin and Rusbult (1995), the Taiwanese samples in the study of Le and Agnew (2001) as well as the Chinese and Japanese population in the study of Apostolou et al. (2021). The model is thus deemed appropriate to be utilized in this study.

## Materials and Methods

### Participants and Procedure

Altogether 1,108 young adults (68.2% females) were recruited using convenient sampling from Malaysia (*n*=444, 57.2% females), Japan (*n*=316, 87.7% females), and India (*n*=348, 64.7% females). The three countries were selected to fulfill the requirement of the grant and based on the availability of collaborators. The mean age was 22.04 (*SD*=3.05) for the overall sample, 22.95 (*SD*=2.73, range=18–32, 1 missing value) for the Malaysian sample, 19.45 (*SD*=1.11, range=18–26) for the Japanese sample, and 23.22 (*SD*=3.24, range=16–35) for the Indian sample. Moreover, 78.5% of the overall sample when surveyed claimed they were not involved in a romantic relationship. All participants gave their consent before answering the online survey. Malaysian participants received a token of five Ringgit Malaysia (USD 1.20), while the Japanese and Indian participants joined the study voluntarily. The Scientific and Ethical Review Committee of the Universiti of Tunku Abdul Rahman (ref: U/SERC/80/2020) reviewed and approved the project.

### Measurements

Both Malaysian and Indian participants answered the measurements in English while the Japanese participants answered the Japanese version of the measurements. For the latter, except for the Relational Mobility Scale (retrieved from http://relationalmobility.org/), all the measurements were first translated by the third author (TN) into Japanese and then back-translated into English by an independent lecturer who is fluent in English and Japanese. Discrepancies were resolved by discussions.

#### Attitudes Toward Singlehood Scale

The nine-item attitudes toward singlehood scale (AtSS) was used to measure individuals’ opinions about singlehood in affective, behavioral, and cognitive dimensions Tan et al., 2021. Each dimension consists of three items. Participants responded to a seven-point Likert scale (1: *strongly disagree*, 7: *strongly agree*). The scores of the nine items were averaged to generate a composite score. The higher score represents a positive attitude toward singlehood; that is, being single is preferable. Sample items were “*I feel comfortable for being single*” and “*Engaging in a romantic relationship is not important*.” The AtSS showed good excellent internal consistency in this study: Cronbach alpha (*α*)=0.931.

#### MacArthur Scale of Subjective Socioeconomic Status

The MacArthur Scale of SSES is a single-item scale for self-reporting relative social-economic status (Adler et al., 2000). Participants indicated their family’s class on a simple ladder ranging from 1 (*lowest*) to 10 (*highest*). A higher score indicates a higher socioeconomic status.

#### Relational Mobility Scale

The 12-item relational mobility scale (RMS) was used to measure individuals’ sense of relational mobility in their community (Yuki et al., 2007). Participants’s tendencies to develop a new relationship in the community was reflected by their agreement on the items describing the people they live with in their respective community using a six-point scale (1: *strongly disagree*; 6: *strongly agree*). Five items (items 4, 5, 7, 9, and 11) were first reversed scored followed by averaging of the 12-item scores. The higher the score, the higher the level of relational mobility. A sample item was “*It is easy for them to meet new people*.” The RMS had satisfactory reliability (*α*=0.781) in this study.

#### Desirability of Control Scale

The desirability of control scale (DCS) comprising 20 items was employed to measure the extent to which the participants desired for control (Burger and Cooper, 1979). They indicated how much the items applied to them on a seven-point scale from 1 (*Does not apply to me at all*) to 7 (*Always applies to me*). The item scores were totaled after reverse-scoring items 7, 10, 16, 19, and 20. The higher scores reflect a higher desire for control. A sample item was “*I enjoy having control over my own destiny*.” The reliability of the DCS in this study was 0.799.

#### Mini-Social Phobia Inventory

Social anxiety was measured by the mini-social phobia inventory (Mini-SPIN) (Connor et al., 2001). Participants indicated the extent to which the statements in the three items described them in the previous week using a five-point Likert scale (0: *Not at all*; 4: *Extremely*). A total score was generated by summing up the item scores. Individuals who reported a higher score are more likely to experience social anxiety. A sample item was “*I avoid activities in which I am the center of attention*.” The Mini-SPIN demonstrated good reliability (*α*=0.817) in this study.

#### Single Item Narcissism Scale

Employing the single item narcissism scale (SINS) to examine narcissism levels, participants indicated their agreement with the item “*I’m a narcissist*” on a seven-point Likert scale (1: *strongly disagree*, 7: *strongly agree*) (Konrath et al., 2014). The higher scorers will exhibit more prominent narcissism in them. The SINS demonstrates good test–retest reliability and validity across 11 independent studies (Konrath et al., 2014).

### Analytic Strategies

The normality of the data was first examined by referring to skewness and kurtosis. Normality is supported for the absolute value of skewness <2 and kurtosis <7 (Kim, 2013). Pearson correlation analysis was then conducted to inspect the variables’ intercorrelation. Finally, hierarchical multiple regression analysis with attitudes toward singlehood as the outcome variable was employed with the following entry sequence: Narcissism and social anxiety entered in Step 1, followed by SSES, relational mobility, and desirability for control entered in Step 2 to examine the extent to which the three predictor variables explain attitudes toward singlehood after statistically controlling for narcissism and social anxiety.

## Results

Table 1 shows the descriptive statistics and interrelationships for the variables. Both skewness and kurtosis values were below 2 indicating that normality assumption was held for all the variables (Kim, 2013). Pearson correlation analysis showed that attitudes toward singlehood were positively associated with social anxiety and desirability of control, negatively associated with SSES, and had no relationship with narcissism and relational mobility. Meanwhile, SSES, relational mobility, and desirability of control were positively associated with one another. Narcissism was found to have a positive relationship with social anxiety and SSES. Finally, social anxiety had a negative relationship with SSES, relational mobility, and desirability of control, respectively.

**Table 1:** Descriptive statistics and correlations for study variables.

Variable	1	2	3	4	5	6
1. Singlehood	1					
2. Narcissism	−0.038	1				
3. Social anxiety	0.148^***^	0.075^*^	1			
4. SSES	−0.066^*^	0.146^***^	−0.183^***^	1		
5. Relational mobility	−0.026	−0.012	−0.083^**^	0.076^*^	1	
6. Desirability of control	0.219^***^	−0.053	−0.172^***^	0.088^**^	0.177^***^	1
*M*	4.38	3.22	5.34	5.64	3.93	90.13
*SD*	1.35	1.59	3.08	1.83	0.61	12.39
Skewness	−0.17	0.23	0.24	−0.12	−0.07	−0.01
Kurtosis	−0.81	−1.06	−0.65	0.12	0.72	−0.01

*N=1,108. Skewness standard error=0.07; Kurtosis standard error=0.15. Singlehood, attitudes toward singlehood; SSES, subjective socioeconomic status; M, mean; SD, standard deviation*.

* *p<0.05*;

** *p<0.01*

*** *p<0.001*.

As planned, the data were submitted to hierarchical multiple regression analysis (see Table 2) to examine the roles of SSES, relational mobility, and desirability of control in attitudes toward singlehood while controlling for the effect of narcissism and social anxiety. Step 1 was found statistically significant, *F*(2, 1,105)=13.79, *p*<0.001, and explained 2.40% of the total variance. Social anxiety (*p*<0.001), but not narcissism, had a significant and positive relationship with attitudes toward singlehood. Similarly, Step 2 was statistically significant, *F*(5, 1,102)=21.83, *p*<0.001, and explained 9.00% of the total variance. Among the five predictors, only social anxiety (*p*<0.001) and desirability of control (*p*<0.001) had significant positive relationships with attitudes toward singlehood. All the variance inflation factor (VIF) values were below 1.10 indicating that multicollinearity was not an issue.

**Table 2:** Hierarchical regression results for attitudes toward singlehood (*N*=1,108).

Variable	*B*	95% CI for *B*		*SE B*	*β*	*R* ^2^	Δ*R*^2^	*LL*	*UL*
Step 1						0.024	0.024^***^
Constant	4.16	3.94	4.37	0.11			
Narcissism	−0.04	−0.09	0.008	0.03	−0.05		
Social anxiety	0.07^***^	0.04	0.09	0.01	0.15		
Step 2						0.090	0.066^***^
Constant	2.33	1.58	3.07	0.38			
Narcissism	−0.03	−0.08	0.02	0.03	−0.03		
Social anxiety	0.08^***^	0.05	0.11	0.01	0.18		
SSES	−0.04	−0.08	0.01	0.02	−0.05		
Relational mobility	−0.12	−0.25	0.01	0.07	−0.05		
Desirability of control	0.03^***^	0.02	0.03	0.003	0.26		

*CI, confidence interval; LL, lower limit; UL, upper limit; and SSES, subjective socioeconomic status*.

*** *p<0.001*.

Despite the sample mainly consisted of single individuals, we are concerned whether the results would be different for single and those in a relationship. Hence, we conducted another hierarchical multiple regression analysis on the two samples, respectively. The results of the single sample (see Table 3) replicated the results of the whole sample. Step 1 was found statistically significant, *F*(2, 867)=3.81, *p*=0.023, and explained 0.90% of the total variance. Social anxiety (*p*=0.007), but not narcissism, had a positive relationship with attitudes toward singlehood. Similarly, Step 2 was statistically significant, *F*(5, 864)=16.95, *p*<0.001, and explained 8.90% of the total variance. Social anxiety (*p*<0.001) and desirability of control (*p*<0.001) had significant positive relationships with attitudes toward singlehood. No other significant relationships were found. Multicollinearity was not identified as the VIF values were not greater than 1.10.

**Table 3:** Hierarchical regression results for attitudes toward singlehood (single individual, *n*=870).

Variable	*B*	95% CI for *B*		*SE B*	β	*R* ^2^	Δ*R*^2^	*LL*	*UL*
Step 1						0.009	0.009^*^
Constant	4.51	4.28	4.73	0.12			
Narcissism	−0.02	−0.08	0.03	0.03	−0.03		
Social anxiety	0.04^**^	0.01	0.07	0.01	0.09		
Step 2						0.089	0.081^***^
Constant	1.79	0.96	2.62	0.42			
Narcissism	−0.01	−0.06	0.04	0.03	−0.01		
Social anxiety	0.06^***^	0.03	0.09	0.01	0.14		
SSES	−0.01	−0.05	0.04	0.02	−0.01		
Relational mobility	0.03	−0.11	0.17	0.07	0.01		
Desirability of control	0.03^***^	0.02	0.03	0.003	0.29		

*CI, confidence interval; LL, lower limit; UL, upper limit; and SSES, subjective socioeconomic status*.

* *p<0.05*;

** *p<0.01*;

*** *p<0.001*.

On the other hand, social anxiety (*β*=0.24, *p*<0.001 in Step 1; *β*=0.23, *p*=0.001 in Step 2) was the only predictor that had a significant relationship with attitudes toward singlehood for the participants in a relationship. Therefore, the results were not presented here for the sake of clarity but are available upon request to the corresponding author.

Moreover, inspired by the reviewer, we reran the hierarchical multiple regression analysis on an exploratory basis to investigate the interaction effects of the three target predictors on the two samples (single vs. those in a relationship), respectively. Specifically, three two-way interaction effects: SSES × Relational Mobility, SSES × Desirability of Control, and Relational Mobility × Desirability of Control were entered in Step 3 before the three-way interaction effect in Step 4. The three target predictors were mean centered prior to the generation of the interaction terms to address the multicollinearity issue.

For the single sample, results showed that Step 3 was significant, *F*(8, 861)=12.47, *p*<0.001, and explained 10.4% of the total variance. Among the three two-way interaction effects, only the interaction of SSES and relational mobility was found significant (see Table 4). However, neither the two-way interaction effects nor the three-way interaction effect was significant in Step 4. On the other hand, none of the interaction effects were significant in Step 3 and Step 4 for those in a relationship. The results were not presented here for the sake of clarity.

**Table 4:** Hierarchical regression results for attitudes toward singlehood with interaction effect (single individual, *N*=870).

Variable	*B*	95% CI for *B*		*SE B*	β	*R* ^2^	Δ*R*^2^	*LL*	*UL*
Step 1						0.009	0.009^*^
Constant	4.51	4.28	4.73	0.12			
Narcissism	−0.02	−0.08	0.03	0.03	−0.03		
Social anxiety	0.04^**^	0.01	0.07	0.01	0.09		
Step 2						0.089	0.081^***^
Constant	6.93	6.17	7.68	0.38			
Narcissism	−0.01	−0.06	0.04	0.03	−0.01		
Social anxiety	0.06^***^	0.03	0.09	0.01	0.14		
SSES	−0.01	−0.05	0.04	0.02	−0.01		
Relational mobility	0.03	−0.11	0.17	0.07	0.01		
Desirability of control	0.03^***^	0.02	0.03	0.003	0.29		
Step 3						0.104	0.014^**^
Constant	6.43	2.91	9.96	1.80			
Narcissism	−0.01	−0.06	0.04	0.03	−0.01		
Social anxiety	0.06^***^	0.03	0.09	0.01	0.14		
SSES	−0.33	−0.75	−0.10	0.22	−0.46		
Relational mobility	−0.11	−0.98	−0.76	0.44	−0.05		
Desirability of control	0.02	0.02	0.06	0.02	0.25		
SSES^*^Mobility	−0.14^**^	−0.22	−0.06	0.04	−0.79		
SSES^*^Control	0.003	−0.001	0.01	0.02	0.33		
Mobility^*^Control	−0.001	−0.01	0.01	0.01	−0.07		
Step 4						0.104	0.000
Constant	6.48	2.91	10.05	1.82			
Narcissism	−0.01	−0.06	0.04	0.03	−0.01		
Social anxiety	0.06^***^	0.03	0.09	0.01	0.14		
SSES	−0.17	−2.08	1.74	0.97	−0.24		
Relational mobility	−0.10	−0.98	0.78	0.45	−0.05		
Desirability of control	0.02	−0.01	0.07	0.02	0.25		
SSES^*^Mobility	−0.10	−0.57	0.37	0.24	−0.57		
SSES^*^Control	0.004	−0.02	0.03	0.01	0.56		
Mobility^*^Control	−0.001	−0.01	0.01	0.01	−0.06		
SSES^*^Mobility^*^Control	0.000	−0.01	0.01	0.003	0.23		

*Values were mean centered except for narcissism and social anxiety. B=unstandardized coefficient; CI=confidence interval; SE=standard error; β=standardized coefficient; LL=lower limit; UL=upper limit; SSES=subjective socioeconomic status; Mobility=Relational Mobility; Control=Desirability of Control*.

* *p<0.05*;

** *p<0.01*;

*** *p<0.001*.

We also employed the model 1 of Hayes (2018) PROCESS macro (ver. 3.3) with 10,000 bootstrap sample and selecting “Mean center for construction of products” in options to probe the significant interaction effect for the single sample. In the first model, SSES was entered as the predictor, attitudes toward singlehood as the outcome variable, relational mobility as the moderator, and social anxiety as the covariate variable. Narcissism was excluded because our hierarchical multiple regression analyses reported above consistently showed no relationship between narcissism and attitudes toward singlehood. After controlling for the effect of social anxiety, (unstandardized coefficient) *B*=0.04, *SE*=0.01, *t*=2.65, *p*=0.008, 95% CI [0.01, 0.07], both SSES and relational mobility did not have a significant relationship with attitudes toward singlehood. However, the interaction effect was found significant: *B*=−0.13, *SE*=0.04, *t*=−2.93, *p*=0.004, 95% CI [−0.21, −0.04]. Further analysis showed that when the relational mobility score is low (i.e., 16th percentiles), SSES had a positive relationship with attitudes toward singlehood, *B*=0.07, *SE*=0.03, *t*=2.15, *p*=0.03, 95% CI [0.006, 0.13]. The relationship between SSES and attitudes toward singlehood was not significant when the relational mobility score was moderate (i.e., 50th percentiles) and high (i.e., 84th percentiles). Similarly, in the second model with relational mobility as the predictor, there was a positive relationship between relational mobility and attitudes toward singlehood, *B*=0.25, *SE*=0.09, *t*=2.68, *p*=0.008, 95% CI [0.07, 0.43] when the SSES level was low but not moderate and high.

## Discussion

This study investigated the antecedent factors of young adults’ attitudes toward singlehood in three Asian countries. The results revealed desirability of control as the key factor of attitudes toward singlehood, even after excluding the effects of narcissism and social anxiety.

Based on the IMC process, SSES, relational mobility, and desirability of control are expected to play a role in attitudes toward singlehood. Our results showed different findings for the singles and those in relationships. On the one hand, none of the three target variables contributed to the attitudes toward the singlehood of people in relationships. On the other hand, mixed findings were found for the singles. The desirability of control was found to have a positive relationship with attitudes toward singlehood. Individuals who reported higher levels of desirability of control are likely to have positive attitudes toward singlehood. The result is in line with the IMC and literature in that people would rather be single to have better control of their lives.

Both SSES and relational mobility did not have a relationship with attitudes toward singlehood as suggested by the IMC. However, our exploratory analysis found a significant interaction effect of SSES and relational mobility. Simple effect analyses showed that when relational mobility was low (vs. moderate and high), SSES was positively associated with attitudes toward singlehood. Put differently, despite being in a social context that has low possibilities in securing a partner, single individuals with higher income do not fuss but view singlehood positively. This could be due to them having sufficient resources to support themselves (i.e., the importance of having a partner is low) and that they wish to continue reaping the benefits of singlehood (e.g., freedom and personal space; Kislev, 2019).

Meanwhile, when SSES was low (vs. moderate and high), there was a positive relationship between relational mobility and attitudes toward singlehood. In other words, with lower financial status than others, single young adults would prefer to stay single to minimize financial burden even if they are living in a social environment that is easy for them to establish a relationship. Taken together, the findings suggest that financial concern is the key to shaping young adults’ attitudes toward singlehood. This is consistent with the literature (e.g., Petrowski et al., 2015; Apostolou et al., 2020) that financial situation is a hindrance to engaging in a relationship.

Overall, our findings contribute to the literature in two ways. Theoretically, our results suggest a prospective direction to expand the IMC process. While the IMC offers explanation to the reasons of developing or keeping a relationship, our findings shed light on the antecedents of attitudes toward single; that is, the reasons of staying single. Moreover, the interaction between SSES and relational mobility suggests that future researchers could consider the conditional effect of satisfaction level, QoA, and investment size. The practical implication of this study is the important role of desirability of control in attitudes toward singlehood. Researchers and practitioners may then design training programs for people concerned about controllability to manage their desire creatively and constructively. This may help them manage their romantic relationships and desirability of control more successfully. Finally, our results suggest that financial concern goes beyond and above desirability of control in shaping attitudes toward singlehood. It is therefore essential for policy-making initiatives (e.g., provide trainings on professional skills) to take place in boosting financial strength in young adults from low-income families. They could then nurture romantic relationships within their capabilities.

Nevertheless, this study consists of some limitations that deserve consideration. First, the difference in the sample size of the two relationship status groups (single vs. in a relationship) posed some difficulties in making a comparison between the two groups. We would recommend future researchers to recruit an equal or comparable sample size to examine if an individual’s relationship status moderates the role of the three predictors in attitudes toward singlehood. In the same vein, it is noteworthy that the RMS measures the level of relational mobility at the social context of respondents rather than their individual level. Although this approach is helpful to minimize the confounding effects of individual characteristics (e.g., personality and attractiveness; Yuki et al., 2007), future researchers may either modify the items and instructions of the RMS or use other measurement tools for participants to report their own relational mobility level. In addition, this study merely focused on three Asian countries. It is therefore inadequate to assume the generalizability of the results to Western countries. Considering that the income level and living cost may vary from one country to another, data collection from different countries is also necessary for exploring the national similarities and differences in the antecedents of the attitudes toward singlehood. Finally, it is noteworthy that this study had collected data from young adults using a cross-sectional design. The findings shall not be interpreted with the lens of causality, though it makes little sense to assume attitudes toward singlehood influence SSES and relational mobility, respectively. This inquiry can be addressed by the future researchers conducting a longitudinal study across young adulthood to adulthood. Such design will not only offer insight into the causality of the relationships but also shed light on the impacts of the antecedent factors on attitudes toward singlehood across time. The latter will be useful in identifying the critical factors of attitudes toward singlehood for the different age groups.

## Conclusion

As there is an increasing trend of young adults prefer staying single to having committed relationships, it is only timely to scrutinize the underlying factors to one’s attitudes toward singlehood. This cross-national study established that the desirability of control is the key factor whereby single individuals tend to believe in the benefits of singlehood that fulfill their needs for controlling their lives. Future researchers interested in singlehood are thus encouraged to focus on distinguishing voluntary single from involuntary single besides identifying the contributors to the two types of singlehood, respectively.

## Data Availability Statement

The raw data supporting the conclusions of this article will be made available by the authors, without undue reservation.

## Ethics Statement

The studies involving human participants were reviewed and approved by Scientific and Ethical Review Committee of the Universiti of Tunku Abdul Rahman. Written informed consent was not provided because participants gave their consent in an e-form before answering the online survey.

## Author Contributions

C-ST and S-MC: conceptualization, design, writing—original draft preparation, and project administration. C-ST, S-MC, TN, and SG: material preparation, data collection, and writing—review and editing. C-ST: analysis. All authors have read and agreed to the published version of the manuscript.

## Funding

The project except for the data collection in India was supported by Sumitomo Foundation FY2019 Grant for Japan-Related Research Projects (No: 198492).

## Conflict of Interest

The authors declare that the research was conducted in the absence of any commercial or financial relationships that could be construed as a potential conflict of interest.

## Publisher’s Note

All claims expressed in this article are solely those of the authors and do not necessarily represent those of their affiliated organizations, or those of the publisher, the editors and the reviewers. Any product that may be evaluated in this article, or claim that may be made by its manufacturer, is not guaranteed or endorsed by the publisher.
